# Sustained remission of rapidly progressive post-transplant immunoglobulin A nephropathy by treatment with tonsillectomy following steroid pulse therapy: a case report

**DOI:** 10.1080/0886022X.2020.1851257

**Published:** 2020-12-16

**Authors:** Aoi Yamashiro, Muneharu Yamada, Yu Kihara, Osamu Konno, Hitoshi Iwamoto, Takashi Oda

**Affiliations:** a Kidney Disease Center, Department of Nephrology and Blood Purification, Tokyo Medical University Hachioji Medical Center, Hachioji, Tokyo, Japan; b Kidney Disease Center, Department of Kidney Transplantation Surgery, Tokyo Medical University Hachioji Medical Center, Hachioji, Tokyo, Japan

Dear Editor,

Post-transplant immunoglobulin A (IgA) nephropathy is frequently observed in patients after renal transplantation for IgA nephropathy, and is associated with an increased risk of graft loss. In particular, crescent formation has been reported to be associated with poor graft prognosis [1]. Although aggressive treatment with methylprednisolone pulses and/or cyclophosphamide is often performed, to date there are no established treatments for post-transplant IgA nephropathy [2,3].

**Figure 1.**
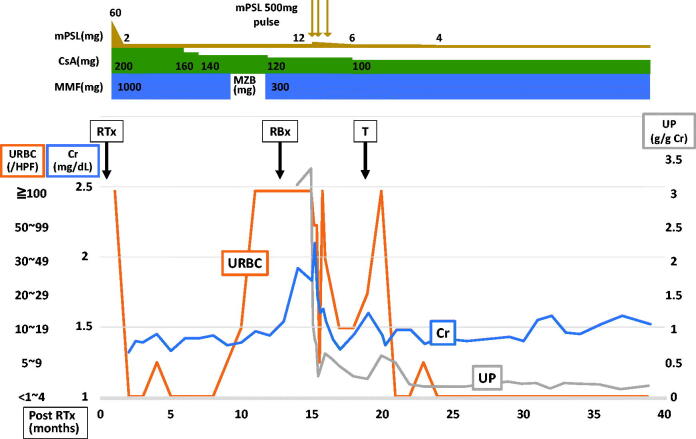
Summary of the clinical course of the patient after renal transplantation. Hematuria appeared at 9 months after renal transplantation, and then serum creatinine level started to increase at 13 months. 3 courses of steroid pulse therapy improved levels of serum creatinine and proteinuria, but middle grade hematuria still persisted at 10–19 RBCs/hpf. We finally performed tonsillectomy at 17 months. His hematuria got worse temporarily but then disappeared completely. The patient eventually achieved long-term complete remission of the urinary abnormalities, and has since then maintained his renal function for 2 years until the most recent follow-up. mPSL: methylprednisolone; CsA: cyclosporine A; MMF: mycophenolate mofetil; MZB: mizoribine; Cr: creatinine; UP: urine protein; RTx: renal transplantation; RBx: renal biopsy; T: tonsillectomy.

A 54-year-old man was referred to our nephrology department because of decline in renal function 13 months after renal transplantation. His proteinuria was first pointed out at a medical checkup at his junior high school, but it was not further examined. When he was 50 years old, he was pointed out as having renal dysfunction and proteinuria at an office medical checkup. As his kidneys were already atrophic, renal biopsy was not performed. His renal function deteriorated, and he started peritoneal dialysis at the age of 52 years. After undergoing peritoneal dialysis for 1.5 years, he underwent a living-donor kidney transplant from his wife. The allograft demonstrated successful engraftment with a stable creatinine level of approximately 1.4 mg/dL with no abnormalities on urinalysis. Protocol biopsies were performed at 3 months and 1 year after transplantation, the pathological analysis of them performed by a clinical laboratory testing company (SRL, Inc., Tokyo, Japan) only reported no evidences of rejection. However, 13 months after renal transplantation, his serum creatinine level started to increase, and was referred to our nephrology department. Urinalysis showed hematuria with more than 100 RBCs/high-power field (hpf), and protein excretion of 1.7 g/day. Laboratory analysis: white blood cell count was 8.66 × 10^3^/µL; hemoglobin concentration, 12.3 g/dL; platelet count, 424 × 10^3^/µL; serum creatinine, 1.83 mg/dL; urea nitrogen, 21.7 mg/dL; C-reactive protein, 4.86 mg/dL; IgG, 1840 mg/dL; IgA, 622 mg/dL; ANCAs, negative. We retrospectively reviewed the patient’s urinary data, and found that he had developed hematuria 9 months after renal transplantation (Figure 1). Therefore, we carefully reevaluated the histological findings of his 1-year protocol biopsy, which was performed after the appearance of hematuria but when his renal function was still stable, and the pathology report indicated no evidences of rejection (Banff ’09 classification: i0, t0, v0, ci0, ct0, cg0, cv0, ptc0, mm0, ah0, aah0, c4d0), and no comments on glomerular changes. Light microscopic analysis of 12 glomeruli sections showed no global sclerosis, mesangial proliferation, endocapillary proliferation or adhesion. However, careful reevaluation of serial sections demonstrated 1 glomerulus with segmental cellular crescent formation and ruptured glomerular tufts (Figure 2(A–C)). Immunofluorescence microscopy showed strong staining of IgA (Figure 2(D)) and C3 (Figure 2(E)) in the mesangial area but was negative for C1q, C4, IgG, or IgM. In addition, electron microscopy showed electron dense deposits in the paramesangial area (Figure 2(F)). From these findings, the pathological condition of the allograft was diagnosed as early and active IgA nephropathy. We started steroid pulse therapy with methylprednisolone (500 mg/day for 3 days) from day 3 of hospitalization after confirming the absence of active infection. The dose of oral methylprednisolone was increased from 4 to 12 mg following the steroid pulse therapy. A single course of steroid pulse therapy led to substantial improvement; in particular, the levels of serum creatinine and hematuria improved substantially (from 2.1 to 1.6 mg/dL, and from >100 to 5–9 RBCs/hpf, respectively). Therefore, a second course of steroid pulse therapy was performed on day 10 of hospitalization. However, the effect was not continuous; hematuria of 10 to >100 RBCs/hpf still continued. We further added a third course of steroid pulse therapy a month after discharge, but his hematuria still persisted at 10–19 RBCs/hpf. Thus, we found that the effect of steroid pulse therapy on the urinary abnormalities of this patient were not continuous but limited. We finally performed tonsillectomy 3 months after discharge, expecting a sustained renoprotective effect. As a result, the patient’s hematuria got worse temporarily but then disappeared completely 3 months after the tonsillectomy. The patient eventually achieved long-term complete remission of the urinary abnormalities, and has since then maintained his renal function for 2 years until the most recent follow-up (Figure 1).

**Figure 2.**
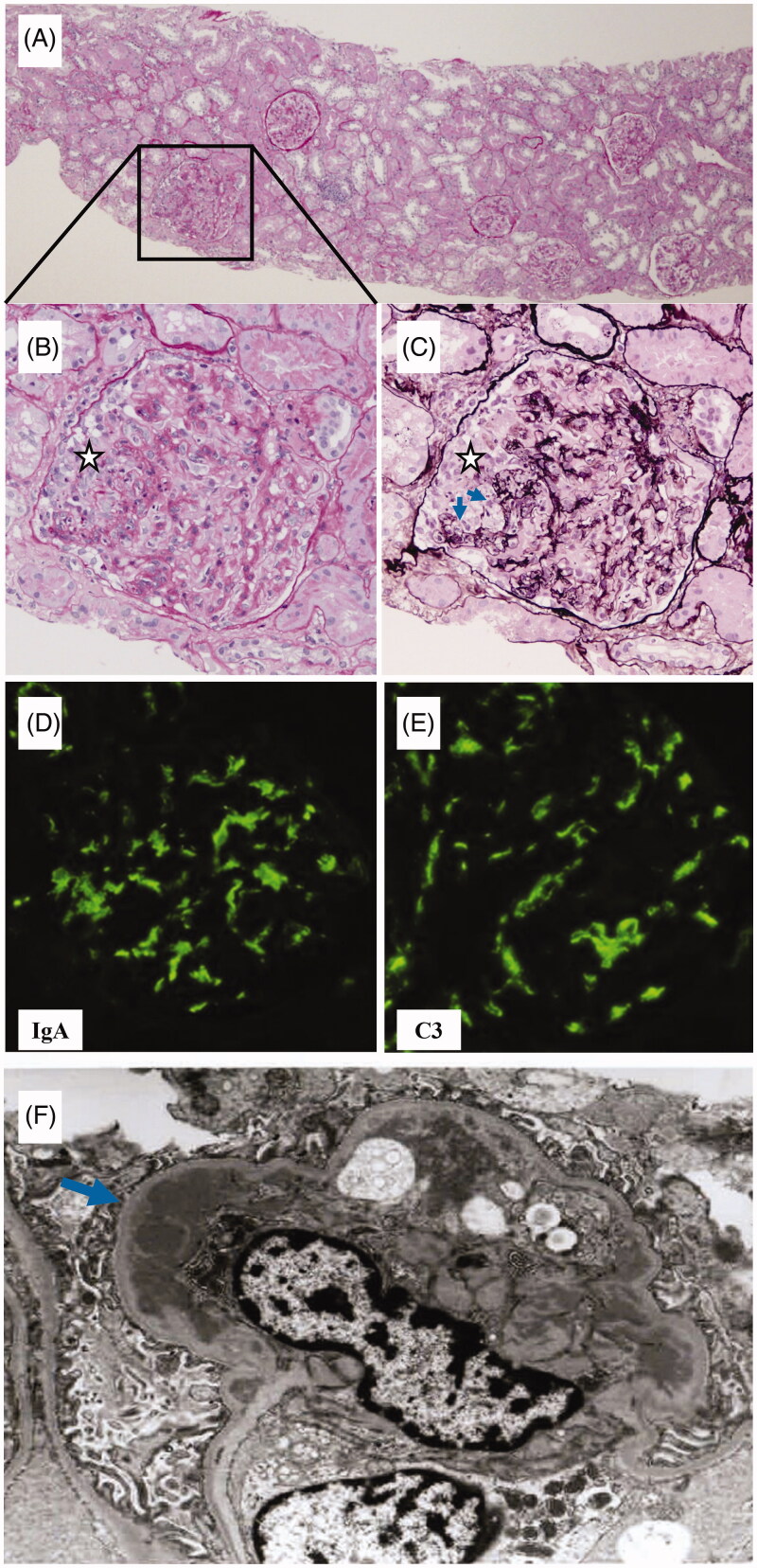
Representative photographs of sections of the 1-year protocol kidney biopsy. (A) Low-power photograph of a periodic acid-schiff (PAS)-stained section showing minor glomerular changes in most of the glomeruli, except for 1 glomerulus with crescent formation (original magnification, ×40). (B) High-power photograph of a PAS-stained section showing a glomerulus with cellular crescent (**⋆**) (original magnification, ×200). (C) High-power photograph of a periodic acid silver-methenamine (PAM)-stained section showing the same glomerulus with rupture of the glomerular capillary walls (arrows) and formation of a cellular crescent (**⋆**) (original magnification, ×200). (D,E) Positive staining for IgA (D) and C3 (E) were observed in the mesangial area of glomeruli by immunofluorescence staining (original magnification, ×200). (F) Electron-dense deposits were observed in the paramesangial area of the glomerulus by electron microscopy (arrow).

Glomerular disease with mesangial IgA deposit is often observed in renal allografts, mainly in 3 conditions, i.e. transmission, recurrent, and *de novo* IgA nephropathy. In this patient, transmission was unlikely, because protocol biopsies on 0-h and 3-month showed no IgA or C3 deposits. It is generally difficult, however, to clearly distinguish recurrence from *de novo* IgA nephropathy in renal allografts. In fact, the presence of urinary abnormalities from adolescence onward, and their recurrence in an early phase after transplantation strongly suggest that this patient had recurrent IgA nephropathy, but complete negation of *de novo* IgA nephropathy is impossible without histological information of the native kidney. Furthermore, it is unclear whether the pathological condition of recurrent and *de novo* IgA nephropathy is different or not. Therefore, such conditions are frequently grouped together under the name of post-transplant IgA nephropathy.

Recent research has highlighted the importance of crescent formation in the pathology of post-transplant IgA nephropathy [2,4]. Furthermore, it is noteworthy that the presence of crescent formation in post-transplant IgA nephropathy has been associated with unfavorable graft prognosis, irrespective of the rate of crescent formation; graft prognosis was significantly unfavorable even in the group with a crescent formation rate of 0% < to ≤ 10% of total glomeruli than in group without any crescent formation [5]. Our patient clinically showed rapidly progressive glomerulonephritis, but pathologically showed minor glomerular abnormalities, except for in 1 glomerulus out of 12 glomeruli, which showed segmental crescent formation. Thus, the pathological lesion of this patient was focal and segmental, and therefore could have easily been overlooked if serial sections had not been checked carefully. However, detecting its existence was very important for predicting graft prognosis, according to the report by Park et al. [5].

Tonsillectomy with or without steroid pulse therapy has been proposed as an effective treatment option in IgA nephropathy of native kidney [6,7]. Regarding post-transplant IgA nephropathy, however, there is no large clinical study analyzing the efficacy of tonsillectomy. There have been reports only with limited number of cases [8]. In our present patient, we initially chose steroid pulse therapy expecting complete remission. Although it was partially effective, middle grade hematuria persisted. The clinical importance of persistent hematuria in IgA nephropathy has recently been demonstrated [9], therefore we performed additional tonsillectomy as a treatment for persistent hematuria. The patient’s hematuria transiently got worse but then completely disappeared after a while. Transient aggravation of hematuria after tonsillectomy is not unusual, and has been reported to occur in about 70% of patients with IgA nephropathy of native kidney. The patient achieved and maintained complete remission of his urinary abnormalities and his renal function. Sustained remission of urinary abnormalities by tonsillectomy in this patient suggests the crucial role of tonsils in the pathogenesis of post-transplant IgA nephropathy.

In summary, we encountered a patient with rapidly progressive post-transplant IgA nephropathy whose active lesion was difficult to determine histologically because of its focal and segmental nature. Performing tonsillectomy after steroid pulse therapy resulted in sustained complete remission. Tonsillectomy may thus be a promising treatment option for patients with rapidly progressive post-transplant IgA nephropathy and persistent hematuria.

## Abbreviations

Ig: immunoglobulin
hpf: high-power field
eGFR: estimated glomerular filtration rate
